# A MEK/PI3K/HDAC inhibitor combination therapy for KRAS mutant pancreatic cancer cells

**DOI:** 10.18632/oncotarget.4538

**Published:** 2015-06-19

**Authors:** Irene Ischenko, Oleksi Petrenko, Michael J. Hayman

**Affiliations:** ^1^ Department of Molecular Genetics and Microbiology, Stony Brook University, Stony Brook, NY, USA

**Keywords:** PDAC, KRAS, MYC, MEK, PI3K

## Abstract

Pancreatic ductal adenocarcinoma (PDAC) is a highly aggressive, metastatic disease with limited treatment options. Factors contributing to the metastatic predisposition and therapy resistance in pancreatic cancer are not well understood. Here, we used a mouse model of KRAS-driven pancreatic carcinogenesis to define distinct subtypes of PDAC metastasis: epithelial, mesenchymal and quasi-mesenchymal. We examined pro-survival signals in these cells and the therapeutic response differences between them. Our data indicate that the initiation and maintenance of the transformed state are separable, and that KRAS dependency is not a fundamental constant of KRAS-initiated tumors. Moreover, some cancer cells can shuttle between the KRAS dependent (drug-sensitive) and independent (drug-tolerant) states and thus escape extinction. We further demonstrate that inhibition of KRAS signaling alone via co-targeting the MAPK and PI3K pathways fails to induce extensive tumor cell death and, therefore, has limited efficacy against PDAC. However, the addition of histone deacetylase (HDAC) inhibitors greatly improves outcomes, reduces the self-renewal of cancer cells, and blocks cancer metastasis in vivo. Our results suggest that targeting HDACs in combination with KRAS or its effector pathways provides an effective strategy for the treatment of PDAC.

## INTRODUCTION

Pancreatic ductal adenocarcinoma, the most common form of pancreatic cancer, is a highly aggressive disease characterized by an insidious onset and low survival rate. About 80% of patients with PDAC present with locally advanced or metastatic disease [1, 2]. The two main obstacles in the treatment of PDAC are late diagnosis and therapy resistance of the tumor. The efficacy of therapies in metastatic cancer is further limited by frequent acquisition of multidrug resistance. Most patients with metastatic pancreatic cancer are considered incurable and rarely survive more than one year [1, 2]. The origins of pancreatic cancer, the feasibility of early detection, and the identity of genes and pathways responsible for the acquisition of malignant (metastatic) phenotype have yet to be determined.

Whole genome sequencing of pancreatic cancers revealed an average of ~45 mutations per tumor, three to six of which are driver gene mutations, while the remaining are passenger mutations that confer no survival advantage [3-6]. The four most frequent mutations in PDAC include KRAS (>90%), CDKN2A (>90%), TP53 (~70%), and SMAD4 (~50%), which are considered to be founder mutations and have been implicated in the metastatic process [3-6]. Pancreatic cancers with high metastatic capacity are further subdivided into two genetic groups: KRAS/TP53 double mutant and KRAS/TP53/SMAD4 triple mutant. These mutations are thought to arise sequentially, resulting in the development of increasingly aggressive cancer phenotypes [7, 8]. Even with this genetic information, unanswered questions remain about critical drivers of metastatic progression, and whether some driver mutations make metastatic cancer a certainty and render it less responsive to treatment. Given the increasing recognition that pancreatic cancers tend to spread early [9], there is an urgent need to quantitatively assess therapy resistance and site-specific outgrowth of disseminated cells. Only with this knowledge will we be able to combat the disease.

These goals have previously been challenging because of the difficulty of detecting small tumor cell populations. To overcome this limitation, we developed a genetically tractable *in vitro* model system to investigate the origins and evolution of pancreatic cancer cells. As a proof of concept, we isolated the main epithelial cell types from which PDAC originates and characterized their propensity to form metastases [10, 11]. In this study, we explore the relative importance of oncogenic KRAS signaling pathways for tumor maintenance and in conferring therapy resistance. Our analysis reveals that oncogenic KRAS dependency can be relinquished in KRAS-initiated tumors, and that some cancer cells can shuttle between the KRAS-dependent (drug-sensitive) and independent (drug-tolerant) states. We further demonstrate that therapeutic targeting of KRAS signaling alone has limited efficacy against PDAC. However, clinically available drugs, used at clinically achievable doses, can be effective against PDAC when co-administered with epigenetic modifiers, such as inhibitors of histone deacetylases. Our data suggest that targeting HDACs in combination with KRAS effector pathways provides an effective strategy for the treatment of PDAC.

## RESULTS

### Pancreatic cancer metastases display morphological and phenotypic heterogeneity

**Figure 1:**
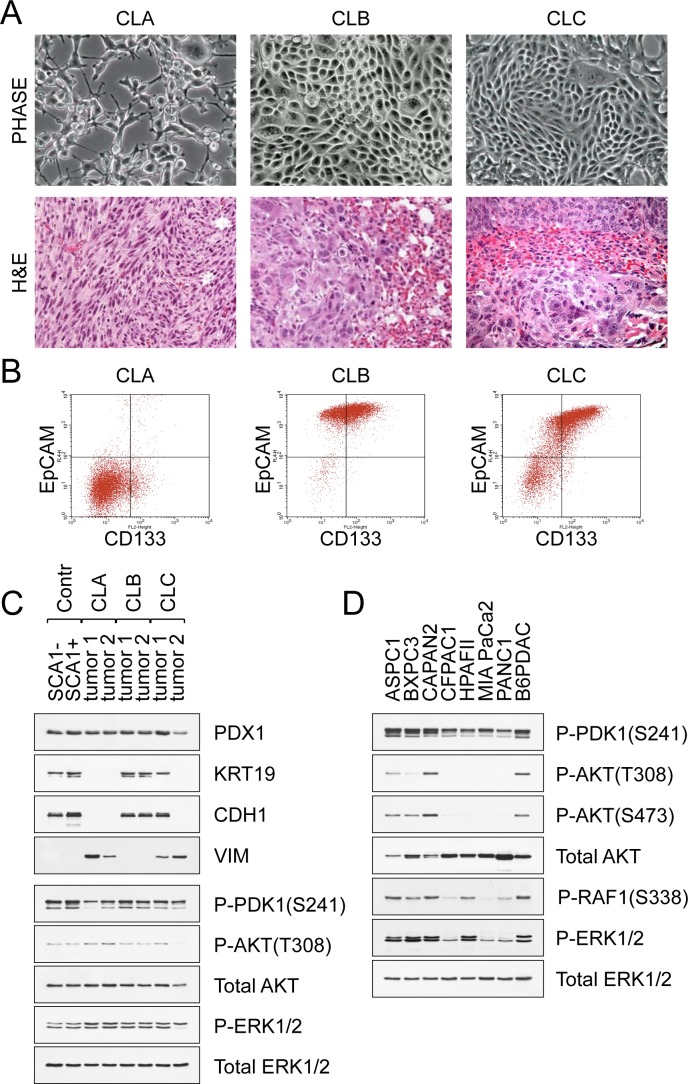
Pancreatic cancer metastases display morphological and phenotypic heterogeneity **A**. Morphological appearance of CLA, CLB and CLC carcinomas derived from KrasG12D p53^KO^ pancreatic cells. Representative H&E-stained sections containing metastatic foci are shown. **B**. FACS analysis of CLA, CLB and CLC carcinomas. **C**. Immunoblot analysis of control pre-tumor cells and representative carcinomas. KRT19 (keratin 19), CDH1 (E-Cadherin), and VIM (vimentin) are shown. ERK1/2 is the loading control. **D**. Western blot analysis of human PDAC cell lines maintained in defined serum-free medium for epithelial cells. A mouse B6-PDAC cell line is shown for comparison.

Using genetically engineered mice carrying KRAS and p53 mutations, we recently identified two main epithelial cell types from which PDAC originates and characterized their propensity to form metastases [10, 11]. The population of less mature cells bears the phenotype of EpCAM+CD24+CD44+SCA1− (referred to as SCA1-) that distinguishes them from a more mature population of EpCAM+CD24+CD44+CD133+SCA1+ cells (referred to as SCA1+) (Fig. S1). The majority of tumors derived from SCA1− cells showed features of undifferentiated (sarcomatoid) carcinoma, whereas the histology of tumors derived from SCA1+ cells exhibited a pattern of well-differentiated adenocarcinoma (Fig. S1). To explore factors contributing to PDAC heterogeneity and therapeutic outcomes, we established clonal cell lines from the respective metastatic foci. The cell lines were assessed for the expression of pancreatic duct specific genes (PDX1, KRT19) and epithelial cell markers (EpCAM, CDH1, CD133). We categorized the cell lines into three groups. Class A cell lines (referred to as CLA) are the pure spindle cell carcinomas exhibiting the EpCAM-CD24+CD44+CD133− surface phenotype (Fig. 1A, 1B). Class B cell lines (CLB) are adenocarcinomas exhibiting a pure epithelial morphology and the EpCAM+CD24+CD44+CD133+ phenotype (Fig. 1A, 1B). Class C carcinomas (CLC) are morphologically heterogeneous and comprise interconvertible EpCAM+CD133+ epithelial and EpCAM−CD133− mesenchymal cells (Fig. 1A, 1B). Based on these features, class C tumors represent reversible epithelial-mesenchymal transition (EMT). Western blot analysis confirmed that CLA carcinomas were Vimentin (VIM) positive, KRT19/CDH1 negative, while CLB carcinomas were VIM negative, KRT19/CDH1 positive (Fig. 1C). Injection of CLA, CLB or CLC cell lines into nude mice led to the development of tumors maintaining the histological appearance of their parental neoplasms (Fig. 1A). CLB clones are representative of the predominant form of human metastatic PDAC [12] and hence we focused our analysis mainly on this cell type.

### Oncogenic KRAS signaling in primary and metastatic PDAC

Signaling through the RAS/MAPK and PI3K pathways plays a causative role in pancreatic carcinogenesis [1, 2]. To assess the contribution of these pathways to PDAC maintenance, we evaluated the growth of the different subtypes in defined serum-free medium for epithelial cells, in the presence of exogenous growth factors, or under non-adherent culture conditions that mimic cancer cell dissemination [10]. Analysis confirmed that the KRAS oncogene activates the MAPK signaling (as assessed by phosphorylated ERK1/2) and PI3K/PDK1 signaling (as assessed by phosphorylated PDK1), but not PI3K/AKT signaling (Fig. 1C). Addition of various growth factors further potentiated MAPK/ERK signaling, and activated AKT in epithelial type CLB carcinomas, but not in mesenchymal type CLA carcinomas (Fig. S2). Similar analysis of human PDAC cell lines also revealed robust phosphorylation of PDK1, but only weak phosphorylation of AKT (AKT-T308 and AKT-S473) (Fig. 1D and Fig. S2). These data suggest that PI3K/PDK1-mediated signaling may play an AKT-independent role in mediating the effects of oncogenic KRAS in pancreatic cancer. We also noted the absence of conspicuous ERK activation in three out of six KRAS mutant PDAC cell lines (CFPAC1, MiaPaCa2 and PANC1) when cultured in serum-free conditions, suggesting that in these cells KRAS oncogenes may no longer maintain their proliferation (Fig. 1D). These results align with previously published data showing that PDAC cell lines harboring KRAS mutations can differ in their dependence on oncogenic KRAS [13-15]. They also help explain why PDAC progression is strongly aided by elements of the tumor microenvironment, including growth factors and cytokines [16-18]. However, the core signaling pathways (RAS/MAPK and PI3K) appear to be valid therapeutic targets for the treatment of PDAC, and these are maintained in our cell system.

### An *in vitro* system to study tumor dormancy and the switch to metastatic growth

**Figure 2:**
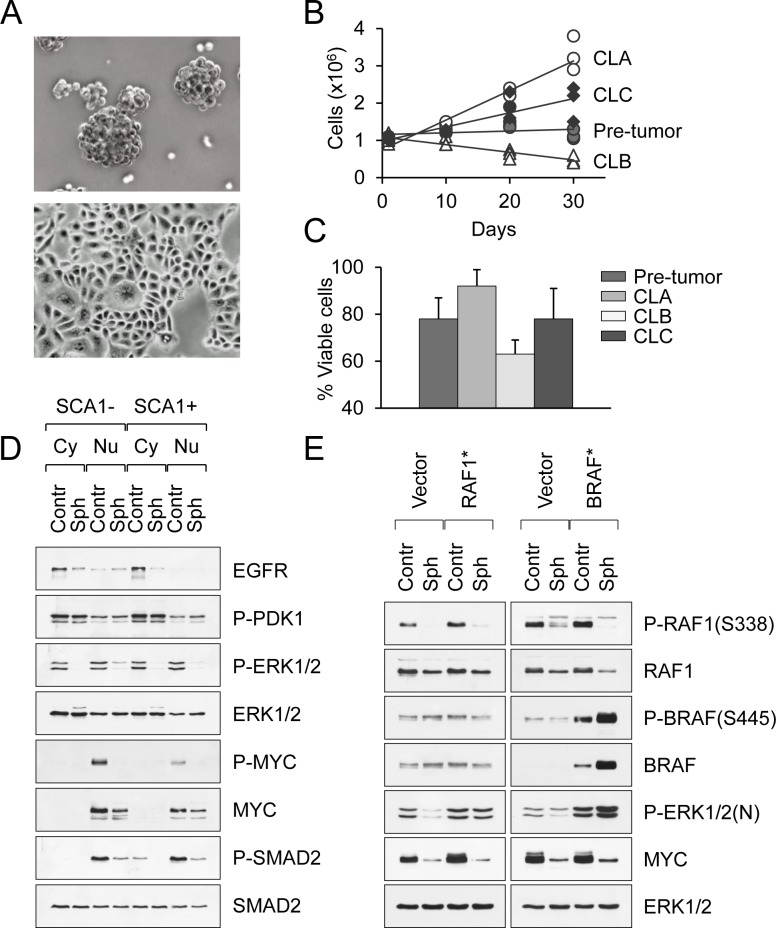
An *in vitro* system to study tumor dormancy and the switch to metastatic growth **A**. Morphological appearance of PDAC cell lines in suspension culture. Spheroids and epithelial sheets are shown. **B**. Long-term survival of primary and tumor-derived PDAC cell lines in non-adherent conditions. **C**. The percentage of viable pancreatic carcinoma cells after 2 weeks in suspension culture. Data are represented as mean ± SD. **D**. Western blot analysis of primary SCA1− and SCA1+ cells maintained in adherent (control) or suspension culture (spheres) for 3 days. Cytoplasmic (Cy) and nuclear (Nu) extracts are shown. **E**. Western blot analysis of CLB carcinoma cells transduced with constitutively active RAF1 (RAF22W) or BRAF (BRAFV600E) mutants and maintained in adherent (control) or suspension culture (spheres).

To mimic cancer cells when they disseminate, we used a 3D non-adherent culture system. An outstanding feature of pancreatic cancer cell lines is their ability to exist in suspension as sphere-like clusters (CLA and CLC carcinomas) or epithelial sheets (CLB carcinomas) (Fig. 2A). These non-adherent conditions shift them to a non-proliferative dormant state, in which they exist for long periods of time (≥1 month) without loss of viability (Fig. 2B, 2C). These changes are coupled with the suppression of MAPK/ERK and SMAD2/3 signaling, yet PI3K/PDK1 signaling is unperturbed (Fig. 2D and Fig. S3). Phosphorylation of RAF1 at S338, which is a critical step in ERK activation, is inhibited in suspended cancer cells (Fig. 2E). Constitutively active RAF1 (RAF22W) and BRAF (BRAFV600E) were both capable to rescue activation of ERK in non-adherent conditions (Fig. 2E). However, even with forced activation of RAF and ERK in suspended cells we were unable to relieve MYC down-regulation and cell growth inhibition (Fig. 2E). Thus, suspension not only inhibits RAF1, but also MAPK signaling downstream of ERK. This dormant state was reversible, as cells from suspensions could readily attach to an appropriate surface and reacquire their malignant phenotype characterized by the persistent activation of the MAPK/ERK/MYC signaling cascade (Fig. 2F and Fig. S3). Hence, we conclude that the growth of pancreatic cancer cells *in vitro* can be suspended (although not completely abrogated) through loss of cell attachment or combined MAPK/ERK/MYC inhibition.

### PDAC cells are acutely susceptible to a MEK/PI3K/HDAC inhibitor combination

**Figure 3:**
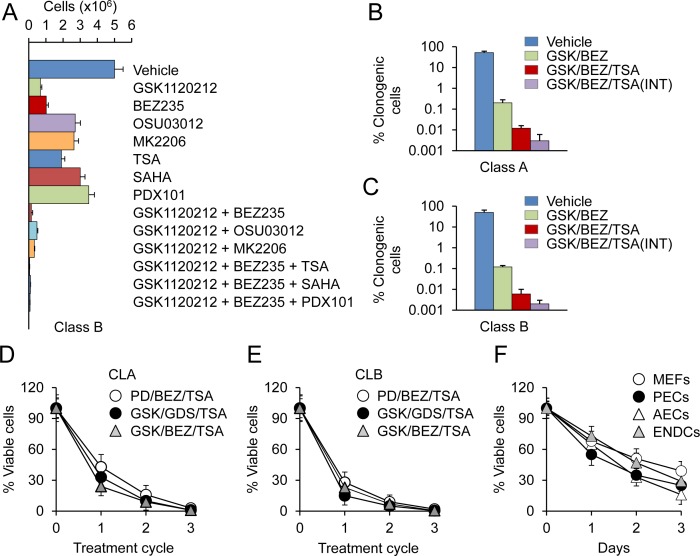
PDAC cells are acutely susceptible to a MEK/PI3K/HDAC inhibitor combination **A**. CLB carcinoma cells were treated for 3 days with the indicated inhibitors as single agents or in combination at a concentration 0.1 μM. Cells were counted by direct counting. Data are represented as mean ± SD. **B.**, **C**. CLA or CLB carcinoma cells were treated for 3 days with GSK and BEZ (GSK/BEZ); GSK, BEZ and TSA (GSK/BEZ/TSA); or three rounds of 3d treatment, each followed by a 3d drug-free period (INT). Clonogenic cells were counted after 4 wks. **D.**, **E**. Tumor spheres derived from CLA or CLB carcinoma cells were subjected to 3 rounds of 3d treatment with MEK (PD0325901 and GSK1120212), PI3K (BEZ235 and GDC0941), and HDAC (TSA) inhibitors at a concentration 0.1 μM. The percentage of viable cells was determined by PI staining. **F**. Primary WT MEFs, p53-null pancreatic epithelial cells (PECs), lung airway epithelial cells (AECs) and WT lung endothelial cells (ENDCs) were treated for 3 days with a combination of GSK/BEZ/TSA at a concentration 0.1 μM. Cells were counted by direct counting.

The above findings led us to assess the therapeutic value of blocking MAPK and PI3K signaling in pancreatic tumor cells. To identify optimal therapeutic strategies, we first screened our tumor cell lines for growth inhibition and cell death after exposure to chemical inhibitors of MEK (PD0325901 and GSK1120212), PI3K (BEZ235 and GDC0941), PDK1 (OSU03012), and AKT (MK2206) at clinically achievable doses of ~ 0.1 μM. Treatment with an inhibitor of a single pathway, e.g. MAPK, led to enhancement of other pathways, as noted by others [19] (Fig. S4). Combined targeting of MEK and PI3K blocked the activation of both the MAPK and PI3K pathways and caused growth inhibition and cell death more effectively than any of the other treatments (Fig. 3A). The cells most susceptible to the induction of apoptosis were CLB carcinomas (Fig. 3A). In contrast, ≥10% of CLA and CLC carcinoma cells remained viable regardless of the intensity or duration of treatment (Fig. S5). Likewise, combined MEK and PI3K inhibition only modestly diminished the viability of the suspended (dormant) tumor cells (Fig. S5). These findings indicated that inhibition of these two KRAS effector pathways was insufficient to cause extensive cell death and prompted us to investigate additional treatment options.

To that end, we treated our cells grown either in 2D or 3D conditions with MEK/PI3K inhibitors in combination with more than 20 other drugs directed against known KRAS effectors, such as the RAF/MAPK, SAPK/JNK and PDK1/AKT pathways, as well as other targets, such as DNA methyltransferases, BET bromodomains and histone deacetylases (Fig. S4). From all of these drugs, PDAC cell lines were found to be acutely susceptible to a MEK/PI3K/HDAC inhibitor combination (Fig. 3A). Thus, combined inhibition of MEK and PI3K decreased proliferation by less than 90%, with only mild induction of cell death. In contrast, combined blockade of MEK/PI3K/HDAC inhibited proliferation by >99% with massive apoptosis (Fig. 3A and Fig. S4). Intermittent treatment with low doses of these drugs further reduced cell survival, as only a small minority of tumor cells (~10^−5^) resumed proliferation after drug withdrawal (Fig. 3B, 3C). These findings align with previous data showing that HDAC inhibitors are able to induce apoptosis in multiple cell types, and that pharmacological inhibition of the MAPK and PI3K pathways enhances the apoptotic effects of HDAC inhibition [20-22]. Among the tested compounds, the strongest cytotoxic effects were obtained with GSK1120212, BEZ235 and trichostatin A (TSA). This drug combination was equally effective in killing proliferating and non-proliferating (dormant) cells (Fig. 3D, 3E), and importantly, cancer cells were more sensitive to drug-induced cell death than normal cells (Fig. 3F). We therefore used this drug combination as a tool to investigate the resistance mechanism(s) of KRAS mutant cancer cells and the feasibility of targeted therapies for pancreatic cancer.

We extended these analyses to a panel of human PDAC cell lines that contain activating KRAS mutations but vary in their KRAS dependency [13, 14]. Combinations of MEK and PI3K inhibitors exhibited marked cytostatic but not cytotoxic effects on all cell lines tested. On the other hand, addition of TSA induced extensive apoptosis in KRAS-dependent AsPC1, HPAFII and BxPC3 cell lines but not in KRAS-independent CFPAC1, MiaPaCa2 and PANC1 cell lines, which were resistant to the induction of cell death even at high concentrations of the drugs (Fig. S6). We also evaluated the effects of inhibitors in a panel of eight human lung cancer cell lines. Four of these cell lines (H23, H358, H727 and A549) have activating KRAS mutations, while other cell lines contain wild-type KRAS and are not RAS-activated (Fig. S6). We found that all lung cancer cells with mutant KRAS were sensitive to BEZ/GSK/TSA inhibition (Fig. S6). Moreover, the addition of TSA induced apoptosis in KRAS-mutant cell lines but not in KRAS wild-type EBC1, PC9, H1650, or HCC366 cell lines, which were less strongly affected (Fig. S6). These data indicate the BEZ/GSK/TSA inhibitor combination is selectively toxic to KRAS-dependent cancer cell lines, regardless of tumor cell type.

### Combined MEK/PI3K/HDAC inhibition prevents lung metastasis *in vivo*

**Figure 4:**
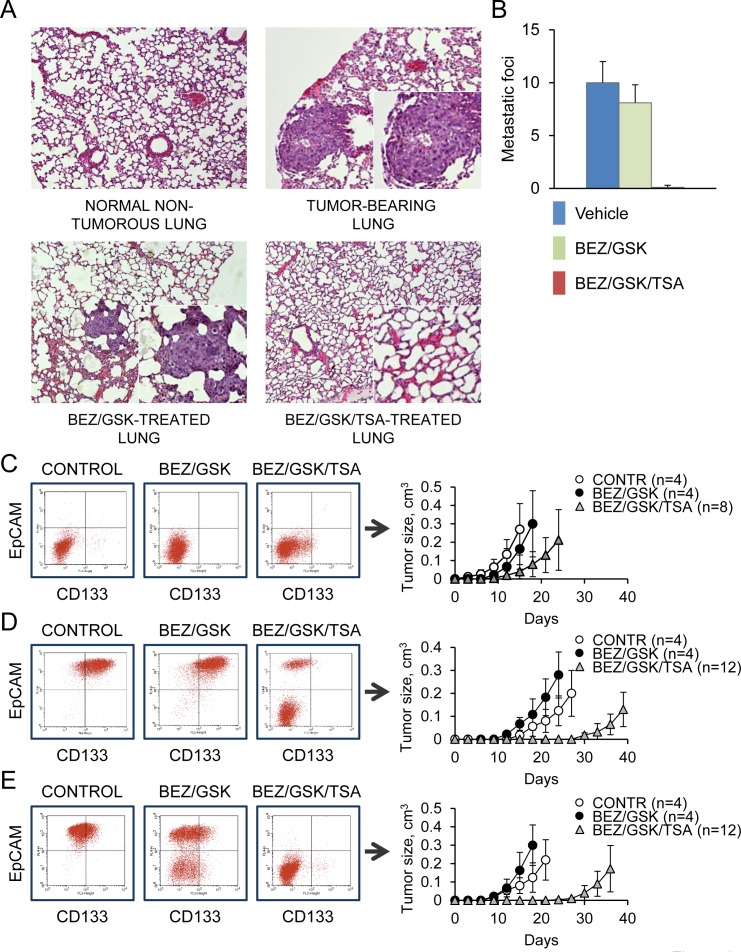
Combined MEK/PI3K/HDAC inhibition prevents lung metastasis *in vivo* **A**., **B**. Development of lung metastases in nude mice injected with CBL carcinoma cells (2×10^4^ cells per injection) and treated with the control vehicle (n= 3), BEZ/GSK (n=3) or BEZ/GSK/TSA (n=4). Data are represented as mean ± SD. Representative H&E-stained sections containing metastatic foci are shown. **C**. CLA carcinoma cells were treated for 2 days with the indicated inhibitor combinations at a concentration 0.1 μM, followed by a recovery period of 1 month. 10^4^ of drug-tolerant cells were analyzed by FACS or injected into nude mice. Tumor latencies are shown. Data are represented as mean ± SD. **D**., **E**. Immature (D) or more mature (E) CLB carcinoma cells were treated with the inhibitors and analyzed as in (C).

We tested the combinations of GSK1120212, BEZ235 and TSA for their potential to suppress metastasis *in vivo*. Nude mice were injected intravenously with 2 × 10^4^ CLB carcinoma cells. Two weeks after the injection of tumor cells, mice were treated daily for 7 days with vehicle or drugs at doses of 1 mg/kg/day and sacrificed 4 weeks later. Earlier work has determined that as few as 10^4^ cells were sufficient to induce lung tumors within 4 weeks after injection [10]. The chosen drug doses were on the low end of effective when used alone or in combination with other drugs (~2 mg/kg daily for GSK1120212; ~40 mg/kg for BEZ235; ~10 mg/kg for TSA) [23-25]. Multiplicity of lung metastatic foci was then determined for each group of mice. Vehicle-treated mice had an average of 10±2 metastatic foci in the lungs per mouse, while mice that received the two drug combination of BEZ/GSK had an average of 8.1±1.7 foci in the lungs (Fig. 4A, 4B). In contrast, there were no observable metastases in mice treated with the BEZ/GSK/TSA inhibitor combination (Fig. 4A, 4B). There were early concerns about possible toxicity of the triple-drug regimen, as MEK and PI3K inhibitors both induce skin rash. As expected, the common side effects associated with dual MEK/PI3K inhibition were skin rash and acneiform eruptions. Remarkably, however, administration of TSA alleviated these adverse effects as we noted no weight loss, toxicity or other side effects during BEZ/GSK/TSA treatment. These results imply that combination therapy with BEZ/GSK/TSA can reduce or prevent cancer metastasis in an *in vivo* model system, and that the drug combination is likely to be reasonably tolerated.

### Transient inhibition of MEK/PI3K/HDAC activity prevents the development of drug resistance

**Figure 5:**
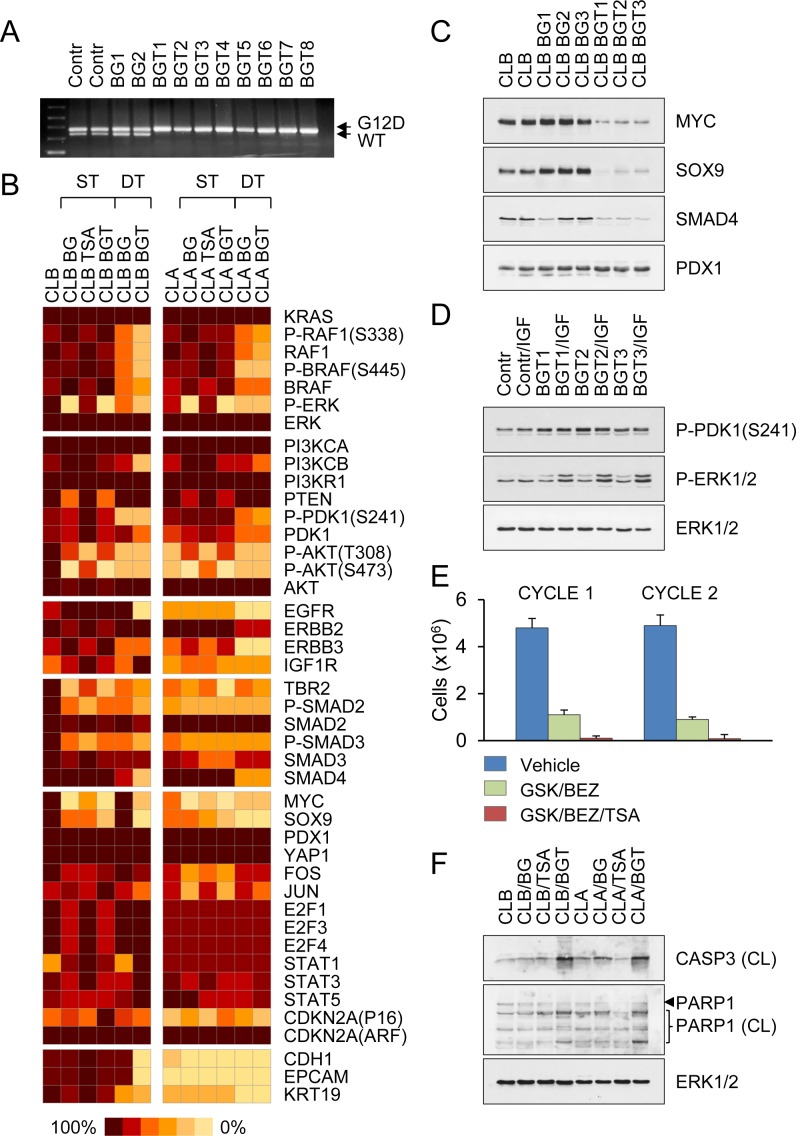
Transient inhibition of MEK/PI3K/HDAC activity prevents the development of drug resistance **A**. Genomic PCR of control-untreated and drug-tolerant CLB carcinoma cells derived after treatment with BEZ/GSK (BG) or BEZ/GSK/TSA (BGT) distinguishes recombined KrasG12D allele from WT KRAS allele by addition of 40 bp in the intronic region. **B**. Schematic heat map showing protein expression values in untreated controls, stressed cells (ST) exposed for 2 days to TSA or combinations of two (BEZ/GSK) or three drugs (BEZ/GSK/TSA), and drug-tolerant (DT) CLB and CLA carcinoma cells that resumed proliferation after drug treatment. Color key for expression levels is shown. **C**. Representative Western blots of BEZ/GSK (BG) or BEZ/GSK/TSA (BGT) drug-tolerant cells derived from CLB carcinomas. **D**. Western blot analysis of drug-tolerant cells shown in (C). Cells were stimulated for 20 min with100 ng/ml of insulin-like growth factor 1 (IGF). **E**. Drug-tolerant CLB carcinoma cells were subjected to consecutive treatment cycles with the indicated inhibitors, followed by a recovery period of 1 month. Similar proportions of tumor of cells were killed with each treatment cycle. **F**. Combined MEK/PI3K/HDAC inhibition enhances apoptosis as evidenced by induction of CASP3 activation and PARP1 cleavage.

Acquired drug resistance is a major problem in cancer therapy. To investigate whether transient inhibition of MEK/PI3K/HDAC activity can lead to the development of drug resistance, we adopted a short-term (2-day) treatment with the drugs (BEZ/GSK/TSA), followed by a recovery period of 1 month. Longer treatment resulted in almost complete elimination of CLB carcinoma cells. We consistently observed that the surviving populations produced slowly proliferating progeny that resembled dormant cells. While the majority of drug-tolerant cells originating from CLA carcinomas retained their aggressive tumorigenic phenotype (Fig. 4C), all drug-tolerant cells originating from CLB carcinomas were markedly distinct from the parental cells, as they lost their epithelial morphology, assuming the features of EMT, and were less tumorigenic compared with controls upon subcutaneous or tail vein injection (Fig. 4D, 4E and Fig. S7). Allele-specific PCR revealed the presence of the mutant (but not of the WT) KRAS allele in all surviving clones examined (Fig. 5A). KRAS expression levels remained essentially unchanged in all of the treatments (Fig. 5B and Fig. S8). However, RAF1 and PI3KCB protein levels were 10-20% of those in untreated controls (Fig. 5B and Fig. S8). The catalytically active, phosphorylated forms of ERK, PDK1 and AKT were reduced accordingly (Fig. 5B and Fig. S8). Most strikingly, these drug-tolerant cells were able to proliferate and grow into tumors in the absence of RAS-induced stabilization of MYC (Fig. 5B, 5C). In a separate screen, we examined drug-tolerant clones arising from CLA carcinomas. We found that nearly all of these drug-tolerant cells had also lost activation of at least one of the two pathways (i.e., MAPK and PI3K) and had less than half of control levels of MYC (Fig. 5B). Gene expression data from public databases (http://www.broadinstitute.org/gsea) were compared to identify gene expression signatures predictive of response to drugs targeting MEK and PI3K, as well as the response to TSA. The genes regulated by RAS/MAPK and PI3K signaling did not overlap with TSA responsive genes, suggesting that MEK, PI3K and HDAC inhibitors do not act similarly on gene expression. However, we observed a modest but significant overlap among MYC target genes and TSA responsive genes, suggesting that TSA may antagonize MYC function (Fig. S8), consistent with the low levels of MYC protein observed after the triple drug treatment. In sum, these drug-tolerant residual cells shunted into an alternative pathway by activating (or repressing) genes that regulate cell survival, differentiation, and transformation.

Because differentiation into cells with mesenchymal-like phenotypes was evident in all cases, we reasoned that the persistent presence of this mesenchymal phenotype is consistent with a non-mutational, and therefore, possibly reversible mechanism of drug tolerance. Indeed, it was reported that proliferating drug-tolerant cells can be drug-sensitized by drug-free passaging [26]. We found that drug-tolerant cells propagated in drug-free media reacquired their original drug sensitivity after ≥30 population doublings. The drug-tolerant phenotype was relinquished due to the reacquisition of KRAS dependency combined with the hypersensitivity to growth factors (e.g., IGF1), as judged by ERK phosphorylation (Fig. 5D). Notably, consecutive treatment cycles, each followed by a recovery period of 1 month, showed that similar proportions of tumor of cells were killed with each treatment cycle (Fig. 5E). Thus, transient MEK/PI3K/HDAC inhibition does not cause resistance to pathway-targeted drugs. However, small populations of cancer cells can shuttle between the KRAS dependent (drug-sensitive) and independent (drug-tolerant) states and thus escape extinction.

### Drug tolerance is randomly acquired by individual cells within the population

The above data imply either that a population of rare pre-existing cells with KRAS independency-conferring mutations can outgrow drug-sensitive cells, or that the drug-tolerant phenotype can emerge de novo in individual cells within the population. To distinguish between these possibilities, we measured phenotypic switching in CLB carcinoma cells that were treated with the combinations of two (BEZ/GSK) or three drugs (BEZ/GSK/TSA). We found that drug-tolerant cells with mesenchymal differentiation but no identifiable epithelial features accumulated gradually, after two weeks of the treatment, with varying efficiencies for two different drug regimens (Fig. S8). We next analyzed phenotypic conversion of individual cells. To that end, CLB carcinoma cells were treated with the GSK/BEZ/TSA inhibitor combination and, after 10 days of recovery, single-cell sorted by FACS based on EpCAM-positive enrichment. At 4 weeks post treatment, we scored 55% of clones as mostly epithelial (EpCAM-positive), 15% as mostly mesenchymal (EpCAM-negative) and 30% as bi-phenotypic (Fig. S9). The preponderance of epithelial or mixed-lineage survivors in this set of experiments is explained by the adopted mild treatment regimen. In sum, although we cannot rigorously exclude the possibility that rare pre-existing mutant cells acquire and then relinquish their KRAS-independent phenotype, our results suggest that KRAS mutant cells can be reprogrammed, at least *in vitro*, and that the process of drug-induced reprogramming can temporarily reduce or abolish their oncogenic KRAS dependency.

### Dormancy is a plausible mechanism of drug tolerance in cancer cell populations

Our results show that therapeutic targeting of KRAS signaling via inhibition of MAPK/PI3K pathways has limited efficacy against PDAC metastasis. The addition of HDAC inhibitors improves the outcome by a factor of at least 100, and just as importantly, only a small proportion of cancer cells (~0.001%) retain the ability to self-renew. Further improving treatment efficacy and safety requires understanding how HDAC and MEK/PI3K inhibition interact to produce a lethal effect, or possibly how HDAC inhibitors change the state of the cells, such that they become more vulnerable to KRAS inhibition. To address these questions, we assessed gene expression profiles of untreated controls, stressed cells (ST), which were exposed either to TSA or combinations of two (BEZ/GSK) or three drugs (BEZ/GSK/TSA), and drug-tolerant cells (DT) that resumed proliferation after drug treatment (Fig. 5B). Analysis of stressed cells revealed acute changes in the MAPK/ERK, PI3K/PDK1 and TGFB/SMAD signaling modules mentioned above (Fig. 5B). Likewise, drug-tolerant cells showed reduced expression of RAF1, BRAF, TBR2, the ERBB family of receptors and MYC (Fig. 5B), suggesting that alterations at these nodes could play a protective role under conditions that are lethal to the majority of the population. We found that the combined use of MEK/PI3K and HDAC inhibitors enhances apoptosis, as evidenced by induction of CASP3 activation and PARP cleavage (Fig. 5F). Moreover, the strong MYC, SOX9, and EGFR effects can also be attributed to the addition of TSA (Fig. 5B). Given that there are no functional equivalents of the *myc* family of proto-oncogenes that can substitute for MYC in control of cell proliferation and malignant transformation [27, 28], and that N-MYC expression is tissue restricted [29], it is tempting to speculate that the dependency of mutant KRAS tumors on MYC function can be circumvented by alternative modules rather than single genes. It was reported that activation of the PI3K pathway [30], loss of CDKN2A [31], or amplifications of the genomic locus containing YAP1, a downstream target in the Hippo signaling pathway and an activator of the AP1 and E2F transcriptional programs, enable bypass of KRAS addiction [32, 33]. However, in our drug-tolerant cells we found no evidence of up-regulation (stabilization) and nuclear accumulation of YAP1, AP1 (FOS and JUN) or E2Fs (E2F1, E2F3, E2F4) (Fig. 5B). CDKN2A expression also remained unchanged (Fig. 5B), suggesting the presence of alternative mechanisms, which lead to a phenotype reminiscent of dormancy, drive recurrent colony formation upon removal of the drugs, and which involve reversible activation and inactivation of three known pathways (MAPK, PI3K, SMAD) under conditions of drug-induced stress.

## DISCUSSION

The mutated KRAS oncogene is found in approximately 90% of cases of PDAC, and generally in about 30% of human cancers [34]. There has been a strong effort to devise strategies targeting KRAS itself or pathways downstream. Although this approach has an immense potential, no successful therapy for treating KRAS-driven cancers has emerged recently. One of the problems with anticancer drugs is their toxicity and narrow therapeutic window. Another is the genetic and phenotypic heterogeneity common to most human cancers. This heterogeneity affects the signaling pathways involved in tumor growth and poses a formidable challenge for cancer treatment [34]. Recently, it was discovered that tumors with KRAS mutations can differ in their dependence on oncogenic KRAS [13, 14]. It follows that, while targeting KRAS or its effector pathways provides a rational mechanism-based approach, a reasonable alternative is to identify pathways that are not directly regulated by KRAS but whose inactivation is lethal in cancer cells that harbor mutant KRAS. The main objective of this study was to explore cell-autonomous vulnerabilities of pancreatic cancer cells with KRAS mutations and identify treatments that kill these cells rather than stop or slow down their growth. Based on our findings we propose that combined targeting of HDACs and key KRAS effector pathways (MEK and PI3K) provides an effective strategy for targeting PDAC.

Our data indicate that therapeutic targeting of KRAS signaling alone via inhibition of the MAPK and PI3K pathways has limited efficacy against PDAC, as it induces cytostatic rather than cytotoxic effects. Because many ongoing trials have adopted co-targeting the KRAS-mediated MAPK and PI3K signaling as the prevalent therapeutic approach, we have explored cancer-specific vulnerabilities associated with epigenetic deregulation. We have investigated the effects of potent MEK inhibitors, such as GSK1120212, the PI3K inhibitors, such as BEZ235, and the HDAC inhibitors, such as TSA, on cell viability and apoptosis induction in metastatic PDAC cell lines maintained in 2D culture (i.e. monolayers in tissue culture), 3D culture (i.e. spheroids in suspension) or *in vivo* as tumor xenografts. Because of their relative specificity toward cancer cells, HDAC inhibitors represent a class of cancer treatment agents that are reasonably well tolerated [35]. The data from our proof-of-principle experiments suggest that targeting HDACs in combination with the major KRAS effector pathways provides an effective strategy for targeting PDAC. Overall, these data indicate that the epigenetic changes established in KRAS mutant cancers makes them insensitive to killing by RAS pathway inhibitors, while disrupting the balance between acetylation and deacetylation sensitizes them to the treatment. The triple drug combination was found to be effective *in vivo* in suppressing metastatic growth while exhibiting no apparent toxic effects compared with the less effective two drug combination. Our study explicitly set out to model clinically relevant drug exposures. Although caution is warranted in extrapolating these results to the human disease insofar as there is no corroborating clinical evidence, the results from this study establish a new paradigm for pancreatic cancer treatment. The results emphasize the potential utility of therapeutic regimens that target the epigenetic state of cancer cells. Questions remain about the type of cell death mechanisms and possible improvement of the treatment.

Genetically engineered mouse models of PDAC have demonstrated that continuous KRAS signaling is required for both progression and maintenance of PDAC [36-38]. However, preclinical trials in mice indicate that while suppression of KRAS signaling prevents tumor initiation and progression, it fails to eradicate established tumors [34, 39]. Our results align with previously published data showing a strong link between tumor cell dormancy, various degrees of EMT and KRAS independence [13, 32, 33]. Our data also imply that the survivors that remain are those rare cells in the population that stochastically move away from dependency on the KRAS activity and gain a growth advantage when the cancer is targeted by the drug combination. The dormant nature of drug-tolerant cells that emerge after the combination drug treatment is supported by their ability to reversibly inactivate MAPK and PI3K signaling under conditions of drug-induced stress. We estimate that pancreatic cancer cells bearing KRAS mutation can maintain the KRAS “independent” phenotype for ≥ 30 population doublings. According to these estimates, each cancer cell will generate ≥ 2^30^ (i.e. ~10^9^) of KRAS-independent offspring. Considering that a tumor reaching the size of 1 cm^3^ is commonly assumed to contain 10^9^ cells or less, this points to the fact that the KRAS-independent phenotype is durable and strong. We know of some genetic alterations, such as CDKN2A loss and YAP1 amplifications [31-33], that can confer a high degree of RAS independence and therapy resistance. However, because these lesions can be excluded in our system, further examination of the effect of MEK/PI3K/HDAC inhibitors in the mesenchymal subsets of PDAC, which are less KRAS dependent, will be important. Notwithstanding these limitations, our results and those reported for other cancer systems [22, 40] suggest that the combined use of epigenetic drugs with agents that target KRAS or its effector pathways can provide a more effective treatment for metastatic cancer.

## MATERIALS AND METHODS

### Mammalian cells and reagents

Parental and metastatic KrasG12D p53^KO^ PDAC cell lines, the latter of which were established from the respective lung and lymph node metastatic foci, were described previously [10, 11]. Unless otherwise specified, cells were grown on gelatinized plates in CnT-17 media (CellnTec). Single cell suspensions were cultured in ultralow attachment 6-well plates (Corning) with CnT-17 media. Human cell lines were cultured in RPMI or DMEM media supplemented with 5% FBS and 1% penicillin/streptomycin, as recommended by ATCC. For long-term cell proliferation assays, cells were seeded into 6-well plates (10^5^ cells per well) and cultured both in the absence and presence of drugs as indicated below. Inhibitors targeting FAK (PF562271), RAF (TAK632), IGF1R (OSI906 and GSK1904529A) (all from ApexBio), MEK (PD0325901 and GSK1120212), PI3K (BEZ235 and GDC0941), PDK1 (OSU03012), AKT (MK2206), HDAC (PXD101, SAHA, TSA) (all from Selleckchem.com) were prepared as 100 μM stocks in DMSO. Cells were treated with various concentrations of the compounds for 3 days, followed by a 1 day drug-free recovery period, and their proliferation was determined by Coulter counter. For intermittent inhibition, cells were subjected to three rounds of 3 day treatment, each followed by a 3 day drug-free period, over the course of 18 days. Cell viability was measured using propidium iodide (PI) staining. We used replication-defective retroviral vectors encoding MYC, constitutively active mutants of Kras4BG12D, RAF122W, and BRAFV600E. Recombinant retroviruses were produced as previously described [10].

### Tumorigenicity in mice

All animal studies were approved by the Institutional Animal Care and Use Committee at Stony Brook University. Nude mice were inoculated subcutaneously or into the tail vein with 10^4^ cells in 100 μl of OPTI-MEM. We defined tumor latencies as the period between injection of tumorigenic cells into mice and the appearance of tumors of ≥1 mm in diameter. The survival end point was a tumor diameter of 1 cm. P ≤ 0.05 was considered statistically significant. Mouse tissue was harvested and processed as described before [10]. Two pathologists read and scored the slides independently. Treatment with GSK1120212 (1 mg/kg), NVP-BEZ235 (10-40 mg/kg), TSA (1-5 mg/kg) or their combination (at the same dose as monotherapy) was started two weeks after tail vein injection with cancer cells. For *in vivo* dosing, drugs were suspended in 0.5% PEG (Sigma) and 0.2% Tween 80 in distilled water. Drugs were administered once a day by intraperitoneal injection for 5 consecutive days. Mice were sacrificed and examined for the growth of metastatic tumors 1 month after administration of drugs.

### Expression analysis

For flow cytometry, cells were lifted with Accutase (Sigma), stained with antibodies to EpCam, SCA1 (Ly-6A/E), CD24, CD44, and CD133 (BD Pharmingen or eBioscince), and analyzed using FACSCalibur (BD) with CellQuest software. For RNA isolation, cells were harvested with TrIzol reagent (Invitrogen). cDNA synthesis for qRT-PCR was performed using SuperScript cDNA synthesis kit (Invitrogen). PCR primers for genotyping and detecting the WT and KrasG12D alleles were as follows: 5′-tccaacacagatgttcttaggctac and 5′-tccgaattcagtgactacagatgtacagag. PCR products were separated on a 2% agarose gel. Successful recombination (single LoxP site) yields a ~340-bp product (~300-bp in WT Kras allele). Western blotting was performed using antibodies against PDX1 (562160, BD), CDH1 (610181, BD), MYC (N-262, Santa Cruz), KRAS (F234), IGF1R (C20, Santa Cruz), PDK1 (3062), P-PDK1 (S241), AKT (9272), P-AKT (T308), P-AKT (S473), P-ERK1/2 (4370), RAF1 (9422), P-RAF1 (S338), BRAF (9433), P-BRAF (S445), KRT19 (3984), SMAD2 (3103), P-SMAD2 (138D4) (all from Cell Signaling), ERK1/2 (05-157, Upstate). Western blotting data for the indicated proteins were quantified using ImageQuant (Molecular Dynamics, Sunnyvale, CA). ERK1/2 was used to normalize protein loading, and blots were displayed as heat maps reflecting gene expression values.

## SUPPLEMENTARY MATERIAL FIGURES


