# Supplementary material for: A MEK/PI3K/HDAC inhibitor combination therapy for KRAS mutant pancreatic cancer cells
Source: Oncotarget. 2015 Jun 19;6(18):15814–27. doi: 10.18632/oncotarget.4538 (PMC4599239; doi:10.18632/oncotarget.4538)
Supplement: Supplementary file 1 [file oncotarget-06-15814-s001.pdf]

A MEK/PI3K/HDAC inhibitor combination therapy for KRAS mutant pancreatic cancer cells

Supplementary Material

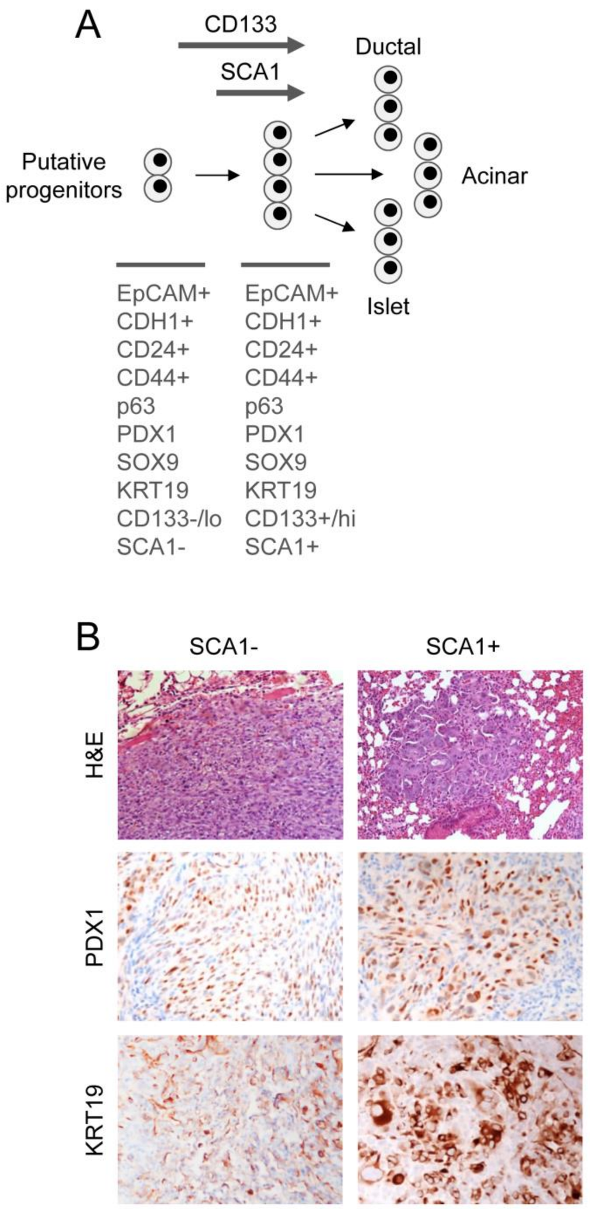

Figure S1. Pancreatic cancer metastases display morphological and phenotypic heterogeneity

A. SCA1 and CD133 expression defines distinct subpopulations of pancreatic cells. The population of presumptive pancreatic progenitors bears the phenotype of EpCAM+CD24+CD44+SCA1- that distinguishes them from more mature EpCAM+CD24+CD44+CD133+SCA1+cells.

B. Representative H&E and IHC-stained sections containing metastatic foci are shown.

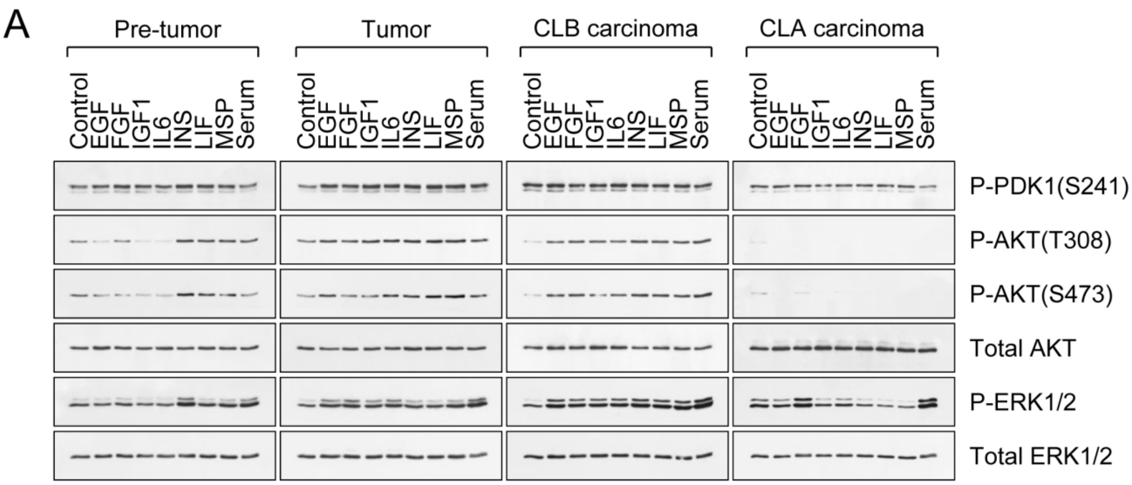

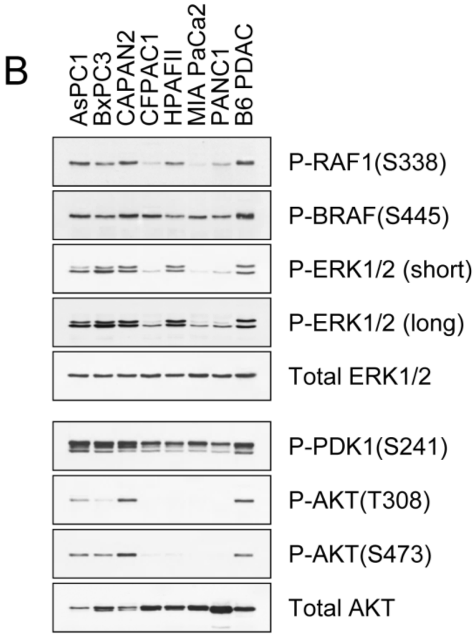

C

Mutational status of human PDAC cell lines used in this study

Cell line	Type	BRAF	CDKN2A	KRAS	P53	SMAD4
AsPC1	PDAC	wt	del	mut	mut	mut
BxPC3	PDAC	mut	wt	wt	mut	del
CAPAN2	PDAC	wt	del	mut	mut	wt
CFPAC1	PDAC	wt	wt	mut	mut	del
HPAFII	PDAC	wt	mut	mut	mut	wt
MiaPACa2	PDAC	wt	del	mut	mut	wt
PANC1	PDAC	wt	del	mut	mut	wt

Figure S2. Oncogenic KRAS signaling in primary and metastatic PDAC

A. Western blot analysis of pre-tumor, tumor-derived and metastatic carcinoma cells maintained in defined serum-free medium for epithelial cells (Contr) and then stimulated for 1h with100 ng/ml of epidermal growth factor, fibroblast growth factor, insulin-like growth factor 1, interleukin 6, insulin, leukemia inhibitory factor or macrophage stimulating protein. Fetal bovine serum was used at a concentration of 2%.

B. Western blot analysis of human PDAC cell lines maintained in serum-free medium for epithelial cells. A mouse B6-PDAC cell line is shown for comparison.

C. KRAS mutation status for each cell line is indicated.

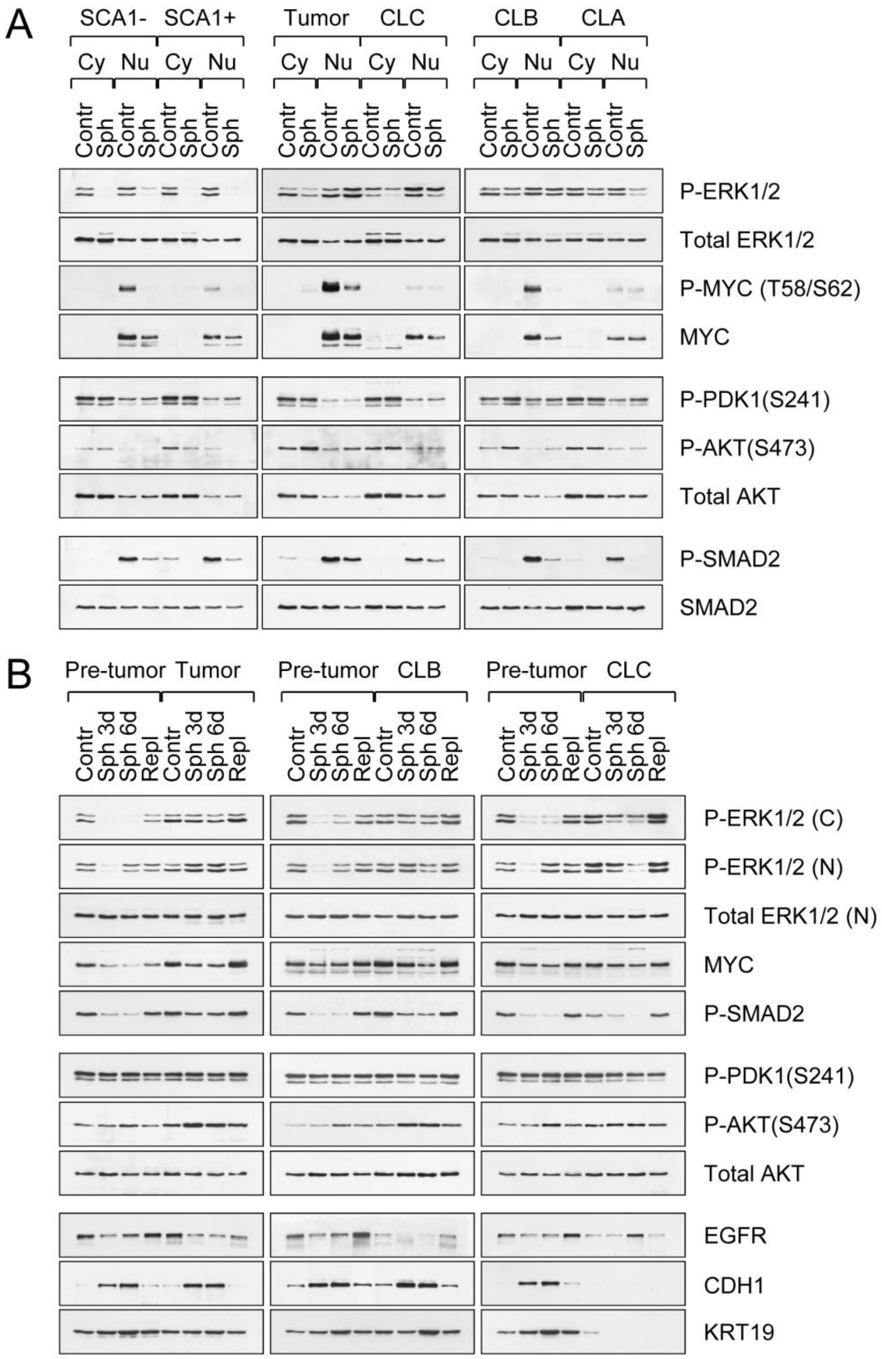

Figure S3. An in vitro system to study tumor dormancy and the switch to metastatic growth

A. Western blot analysis of pre-tumor, tumor-derived and metastatic carcinoma cells maintained in adherent (control) or suspension culture (spheres) for 3 days. Cytoplasmic (Cy) and nuclear (Nu) extracts are shown.

B. SCA1- and SCA1+ cells maintained in suspension culture (spheres) retain adhesive capacity and, when replated on tissue culture dishes, can reacquire their malignant phenotype.

A

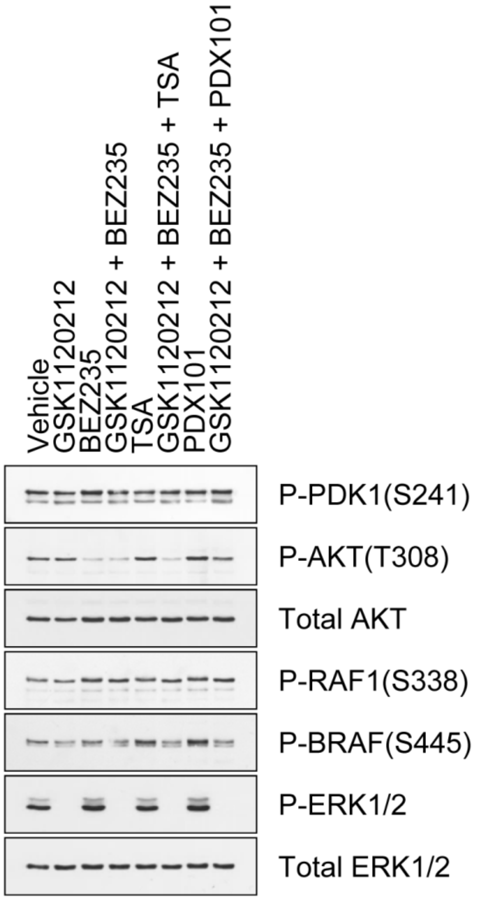

B

	Drug	Target
RAF/MEK/ERK	AZ628	RAF
	TAK632	RAF
	PD0325901	MEK
	PD184352	MEK
	GSK1120212	MEK
PI3K/AKT	BEZ-235	PI3K/mTOR
	GDC-0941	PI3K
	OSU-03012	PDK1
	MKK-2206	PKB/AKT
RTK	Erlotinib	EGFR
	Erbitux	EGFR
	OSI-906	IGF-1R/IR
	GSK1904529A	IGF-1R/IR
Other	PF562271	FAK
	FRAX486	PAK
	SP600125	JNK
	SB431542	ALK5/ALK4
	Vidaza	DMT
	Decitabine	DMT
	JQ1	BRDT
	TSA	HDAC
	SAHA	HDAC
	PDX101	HDAC

Figure S4. Drugs tested in the panel of pancreatic cancer cell lines

A. Western blot analysis of CLB carcinoma cells treated with the indicated inhibitors at a concentration 0.1 μM for 24 hrs.

B. Drugs tested in the panel of pancreatic cancer cell lines.

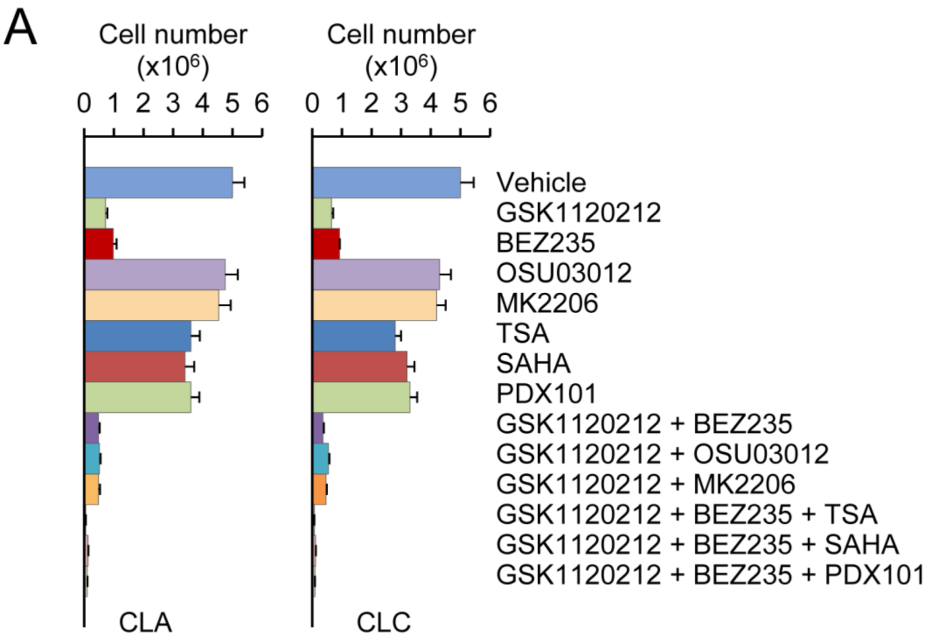

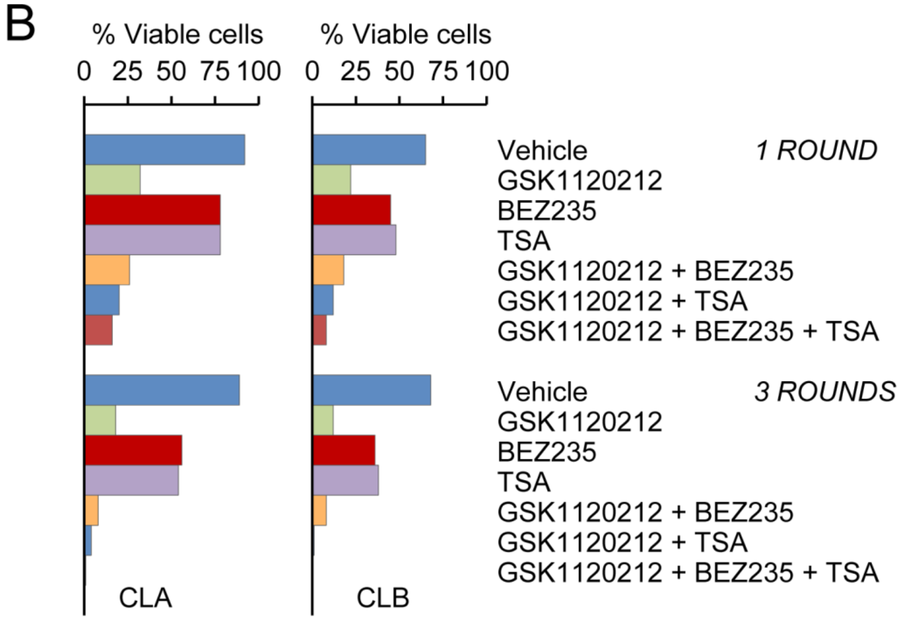

Figure S5. PDAC cells are acutely susceptible to a MEK/PI3K/HDAC inhibitor combination A. CLA and CLC carcinoma cells were treated for 3 days with the indicated inhibitors as single agents or in combination at a concentration 0.1 μM. Cells were counted by direct counting. Data are represented as mean ± SD.

B. Tumor spheres derived from CLA or CLB carcinoma cells were treated for 3 days with the indicated inhibitors as single agents or in combination at a concentration 0.1 μM; or three rounds of 3d treatment, each followed by a 3d drug-free period. The percentage of viable cells was determined by PI staining.

A

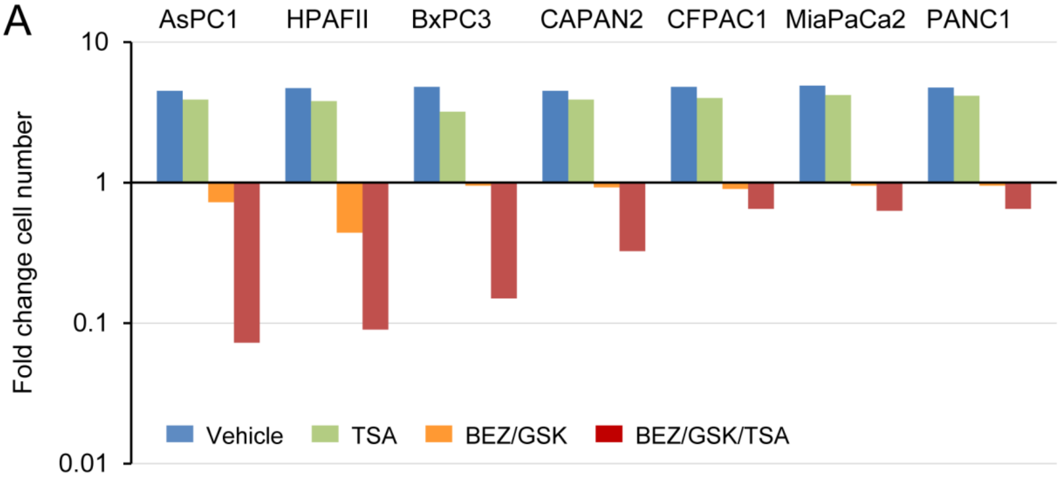

B

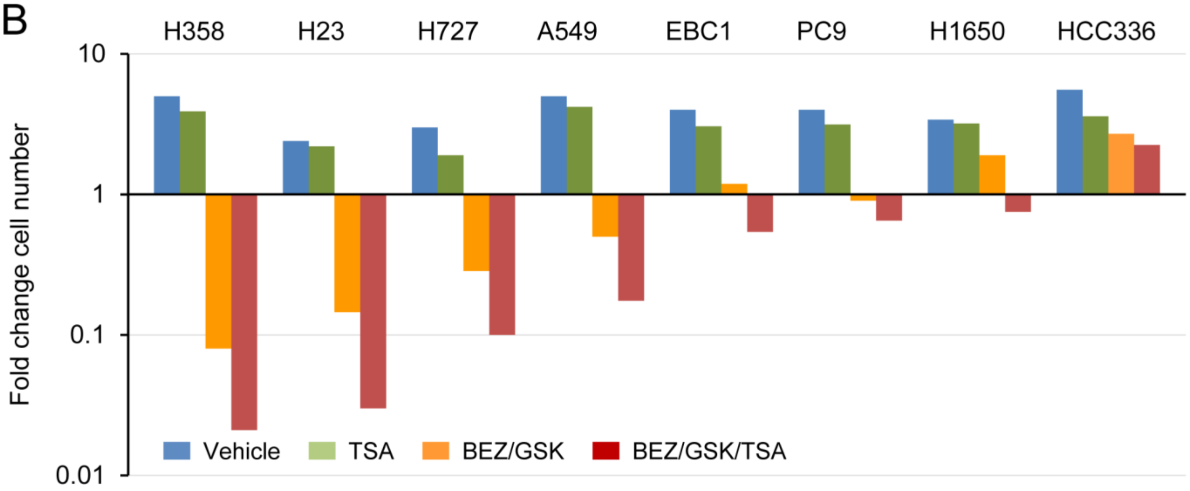

C

Cell line	CDKN2A	EGFR	KRAS	MET	PIK3	P53
H23	wt	wt	mut	wt	wt	mut
H358	wt	wt	mut	wt	wt	del
H727	wt	wt	mut	wt	wt	mut
A549	del	wt	mut	wt	wt	wt
EBC1	wt	wt	wt	amplif	mut (PIK3R2)	mut
PC9	nk	mut	wt	nk	nk	nk
H1650	wt	mut	wt	wt	mut (PIK3C2)	mut
HCC366	wt	wt	wt	wt	mut (PIK3R1)	mut

nk, not known; wt, wild-type.

Figure S6. MEK/PI3K/HDAC inhibitors are selectively toxic to KRAS-dependent cancer cells

Human PDAC cell lines (**A**) or NSCLC cell lines (**B**) were treated for 3 days with the indicated inhibitors at a concentration 0.1 µM. Cells were counted by direct counting. KRAS mutation status for each cell line is indicated (**C**).

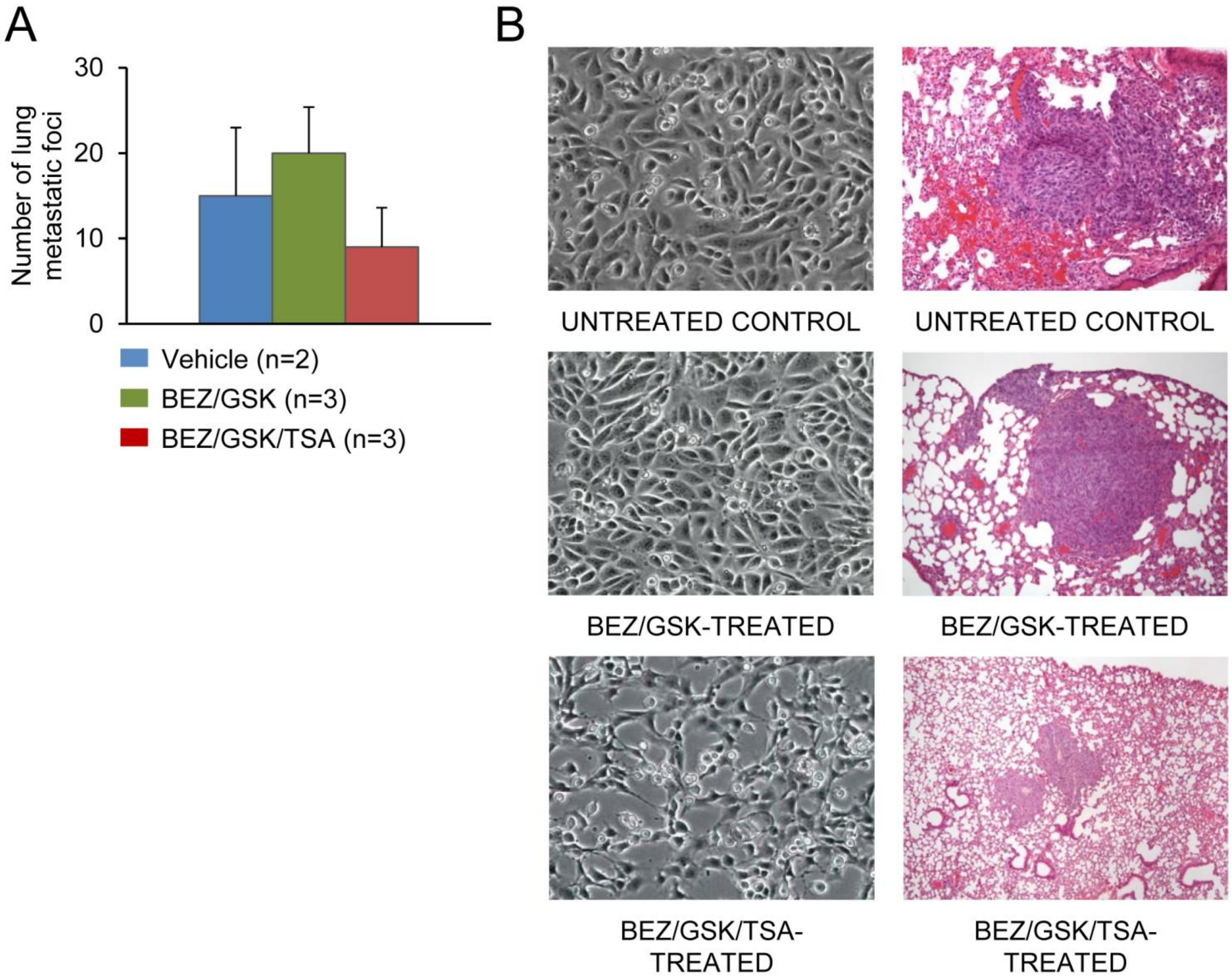

Figure S7. Transient inhibition of MEK/PI3K/HDAC activity prevents the development of drug resistance

A. Left panels: morphological appearance of CLB carcinoma cells treated with BEZ/GSK or BEZ/GSK/TSA inhibitors. Drug-tolerant cells were maintained in drug-free medium for 1 month. Right panels: representative H&E sections containing metastatic foci (arrows) derived from these cells.

B. Development of lung metastases in nude mice by drug-tolerant CLB cell lines (10^4 cells per injection). Data are represented as mean ± SD.

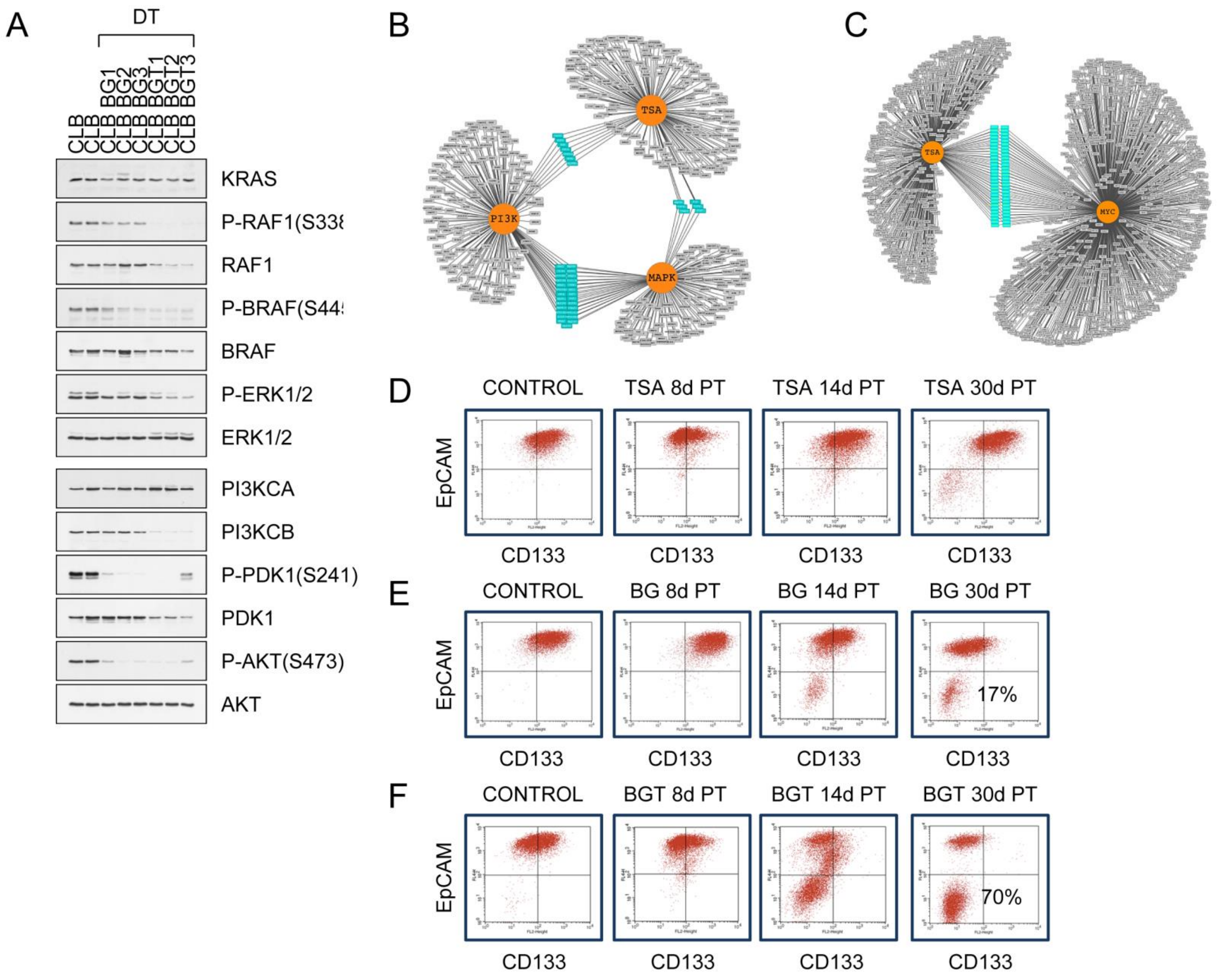

Figure S8. Drug tolerance is randomly acquired by individual cells within the population

A. Representative Western blots of BEZ/GSK (BG) or BEZ/GSK/TSA (BGT) drug-tolerant cells derived from CLB carcinomas.

B, C. Overlap between gene sets regulated by the RAS/MAPK, PI3K and MYC pathways with the TSA responsive genes.

D-F. CLB carcinoma cells were treated for 2 days with the indicated inhibitor combinations at a concentration 0.1 μM, followed by a recovery period of 1 month. 10^4 of drug-tolerant cells were analyzed by FACS.
